# Fabrication of Li_4_Ti_5_O_12_-TiO_2_ Nanosheets with Structural Defects as High-Rate and Long-Life Anodes for Lithium-Ion Batteries

**DOI:** 10.1038/s41598-017-03149-2

**Published:** 2017-06-07

**Authors:** Hui Xu, Jian Chen, Yanhuai Li, Xinli Guo, Yuanfang Shen, Dan Wang, Yao Zhang, Zengmei Wang

**Affiliations:** 1 Jiangsu Key Laboratory of Advanced Metallic Materials, School of Materials Science and Engineering, Southeast University, Nanjing, 211189 China; 2 State Key Laboratory for Mechanical Behavior of Materials, Xi’an Jiaotong University, Xi’an, 710049 Shaanxi China

## Abstract

Development of high-power lithium-ion batteries with high safety and durability has become a key challenge for practical applications of large-scale energy storage devices. Accordingly, we report here on a promising strategy to synthesize a high-rate and long-life Li_4_Ti_5_O_12_-TiO_2_ anode material. The novel material exhibits remarkable rate capability and long-term cycle stability. The specific capacities at 20 and 30 C (1 C = 175 mA g^−1^) reach 170.3 and 168.2 mA h g^−1^, respectively. Moreover, a capacity of up to 161.3 mA h g^−1^ is retained after 1000 cycles at 20 C, and the capacity retention ratio reaches up to 94.2%. The extraordinary rate performance of the Li_4_Ti_5_O_12_-TiO_2_ composite is attributed to the existence of oxygen vacancies and grain boundaries, significantly enhancing electrical conductivity and lithium insertion/extraction kinetics. Meanwhile, the pseudocapacitive effect is induced owing to the presence of abundant interfaces in the composite, which is beneficial to enhancing specific capacity and rate capability. Additionally, the ultrahigh capacity at low rates, greater than the theoretical value of spinel Li_4_Ti_5_O_12_, may be correlated to the lithium vacancies in 8a sites, increasing the extra docking sites of lithium ions.

## Introduction

Large-scale energy storage and renewable energy storage have not only provided better prospects for the applications of lithium-ion batteries (LIBs), but also put forward higher requirements on their energy density, rate capability, cycle life and safety^1–3^. However, conventional graphite anode in LIBs suffers from safety hazards due to their low operating voltage which can cause decomposition of the electrolyte and formation of the solid electrolyte interface (SEI) film. Serious safety problems may occur due to the formation of lithium dendrites, especially at a high-rate and long cycle condition^4^. Spinel Li_4_Ti_5_O_12_ (LTO) is one of the most favored anode materials due to its excellent cycle stability (a consequence of almost negligible volume change during the Li^+^ insertion/extraction processes) and improved safety resulting from its higher insertion voltage (~1.55 V *vs* Li^+^/Li)^5, 6^. However, its low electrical conductivity (~10^−13^ S cm^−1^) and poor lithium-ion diffusion coefficient (~10^−13^ cm^2^ s^−1^) limit its wide use^7, 8^.

A variety of strategies, therefore, have been developed to improve the electrochemical performance of LTO, such as reducing the particle size^9^, doping with foreign atoms^10, 11^ and coating with carbon materials^12^. However, as demonstrated in previous studies, it still remains a great challenge to concurrently obtain the LTO anode materials with both high energy density and high power density.

Recently, research on structural imperfection is very active in the solid-state chemistry field^13^. For example, oxygen vacancy has been recognized to improve the electronic conductivity of electrode materials^14, 15^. Tong *et al*.^16^ prepared the oxygen-deficient α-Fe_2_O_3_ nanorods through thermal decomposition of FeOOH under N_2_ atmosphere, showing substantially enhanced electrochemical performance compared to that of pristine α-Fe_2_O_3_ nanorods prepared in air. Lu *et al*.^17^ reported the oxygen-deficient MnO_2_ nanorods prepared by a simple hydrogenation treatment, delivering significantly improved electrochemical performance than the untreated MnO_2_ electrode, and yielding a large areal capacitance of 0.22 F cm^−1^ with excellent rate capability and cycling stability.

On the other hand, grain boundary can provide fast lithium ion insertion/extraction as the diffusion of lithium ion along the grain boundaries can be orders of magnitude faster than bulk diffusion in the grains, as illustrated in these literatures^18, 19^. Therefore, it is very much expected that if the materials are rich in grain boundaries, they will possess excellent lithium transport and storage properties. To increase grain boundary density with large interfacial areas, it would be wise to choose binary or multinary compounds rather than single compound. Rahman *et al*.^20^ synthesized carbon coated Li_4_Ti_5_O_12_-TiO_2_ composites with high grain boundary density, exhibiting a high capacity of 166 mA h g^−1^ at 0.5 C (1 C = 175 mA g^−1^), good cycling stability, and excellent rate capability. Shen *et al*.^21^ developed a versatile hydrothermal route to fabricate porous LTO/rutile-TiO_2_ nanosheet arrays, delivering a high initial discharge capacity of 184.6 mA h g^−1^ at 200 mA g^−1^ and possessing excellent electrochemical stability with only 8.3% loss of specific capacity at 1 A g^−1^ after 1000 cycles.

Here, we report on a new route to synthesize a LTO-TiO_2_ composite with structural defects. Our purpose is to combine the advantages of both oxygen vacancy and grain boundary to improve the electrochemical performance of LTO. The strategy in this study shows multiple advantages: (1) high concentration of oxygen vacancy enhances the electrical conductivity of electrode materials, (2) grain boundaries, which usually act as channels to facilitate lithium ion diffusion towards the internal of bulk materials, enhance lithium insertion/extraction kinetics, (3) extra lithium ions can be stored in the structural defects including grain boundaries and lithium vacancies, significantly improving the specific capacity, (4) the well-defined TiO_2_ scattering on the surface of LTO suppresses the stacking of LTO nanosheets, therefore shortening the path length for lithium ion transportation. The introduction of structural defects obviously enhances lithium storage properties of the LTO-TiO_2_ composite in terms of ultrahigh specific capacity (182.1 mA h g^−1^ at 1 C, 1 C = 175 mA g^−1^), remarkable rate capability (170.3 mAh g^−1^ at 20 C, 168.2 mAh g^−1^ at 30 C) and long cycle life (161.3 mAh g^−1^ remaining after 1000 cycles at 20 C). In addition, the present strategy may provide a new idea to develop high-performance electrode materials for LIBs.

## Results

The novel LTO-TiO_2_ composite (denoted as LTO-RT-AT) was synthesized using a facile hydrothermal process with subsequent calcination. For a comparative purpose, pure LTO and LTO-rutile TiO_2_ composite (denoted as LTO-RT) were also prepared by changing the molar ratios of Li/Ti in the precursor solution. The detailed experimental processes are presented in the methods section.

**Figure 1:**
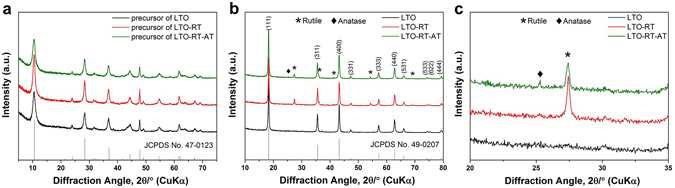
(**a**) XRD patterns of the precursors of pure LTO, LTO-RT and LTO-RT-AT, (**b**) XRD patterns of the three samples after calcination at 600 °C for 6 h in air, (**c**) partially enlarged XRD patterns from 20° to 35° in (**b**).

The X-ray diffraction (XRD) patterns of the precursors of pure LTO, LTO-RT and LTO-RT-AT are shown in Fig. 1a. It is clear that all characteristic diffraction peaks of these precursors are consistent with those of orthorhombic (Li_1.81_H_0.19_)-Ti_2_O_5_·2H_2_O (JCPDS No. 47–0123). Figure 1b shows the XRD patterns of the three samples after calcinated at 600 °C for 6 h in air. The phase combination of the products show strong dependence on the molar ratio of Li/Ti. With a ratio of 4.5:5, the pure LTO was obtained in the final product, all diffraction peaks of the product correspond to the (111), (311), (400), (331), (333), (440), (531), (533), (622) and (444) planes of well-crystallized spinel LTO (JCPDS No. 49–0207). With a decrease in the molar ratio of Li/Ti to 4.2:5 (LTO-RT-AT), two weak peaks at 25.26° and 27.39° arise in Fig. 1c, which can be indexed as the typical peaks of anatase-TiO_2_ and rutile-TiO_2_^22, 23^, respectively, indicating a ternary-phase composite consisting of dominant LTO and a low amount of rutile-TiO_2_ and anatase-TiO_2_ was obtained. When the ratio further drops to 4:5 (LTO-RT), the peak of anatase-TiO_2_ completely disappears and the peaks of rutile-TiO_2_ increase further, indicating that a dual-phase composite including LTO and rutile-TiO_2_ was obtained. On the basis of the Scherrer equation, the average grain sizes of pure LTO, LTO-RT and LTO-RT-AT are evaluated to be 32.6, 31.1 and 26.5 nm, respectively. This indicates that the LTO-RT-AT exhibits the smallest grain size.

The XRD patterns of the calcinated samples with Li/Ti molar ratio of 4.2:5 at 400 °C (LTO-AT-400) and 500 °C (LTO-AT-RT-500) are shown in Fig. S1a (Supporting Information). It is found that the LTO-AT-400 exhibits the almost pure LTO phase with a tiny broad peak at ~25.3°. For LTO-AT-RT-500, not only the peak at 25.3° becomes shaper, but also additions peaks at 37.3° and 48.1° appear. All these three peaks can be assigned to the three strongest peaks of anatase-TiO_2_^22^. Meanwhile, the peaks of rutile-TiO_2_ can be observed at 27.5 and 54.3°^24^. These observations confirm the existence of the TiO_2_ phases (anatase and rutile), and a phase transition from anatase to rutile could start when the calcination temperature exceeds 400 °C. Thermogravimetric (TG) and differential scanning calorimetry (DSC) results of the precursor of LTO-RT-AT are shown in Fig. S1b. There are two major weight-loss regions. The first weight loss occurred between 130 and 250 °C due to desorption of crystal water from (Li_1.81_H_0.19_)-Ti_2_O_5_·2H_2_O. The second weight loss occurred from 250 to 500 °C as (Li_1.81_H_0.19_)-Ti_2_O_5_ was transformed into LTO^25^. In the DSC curve, a broad endothermic peak at ~447 °C is found and this could be related to the conversion of anatase-TiO_2_ to rutile-TiO_2_ as mentioned above.

**Figure 2:**
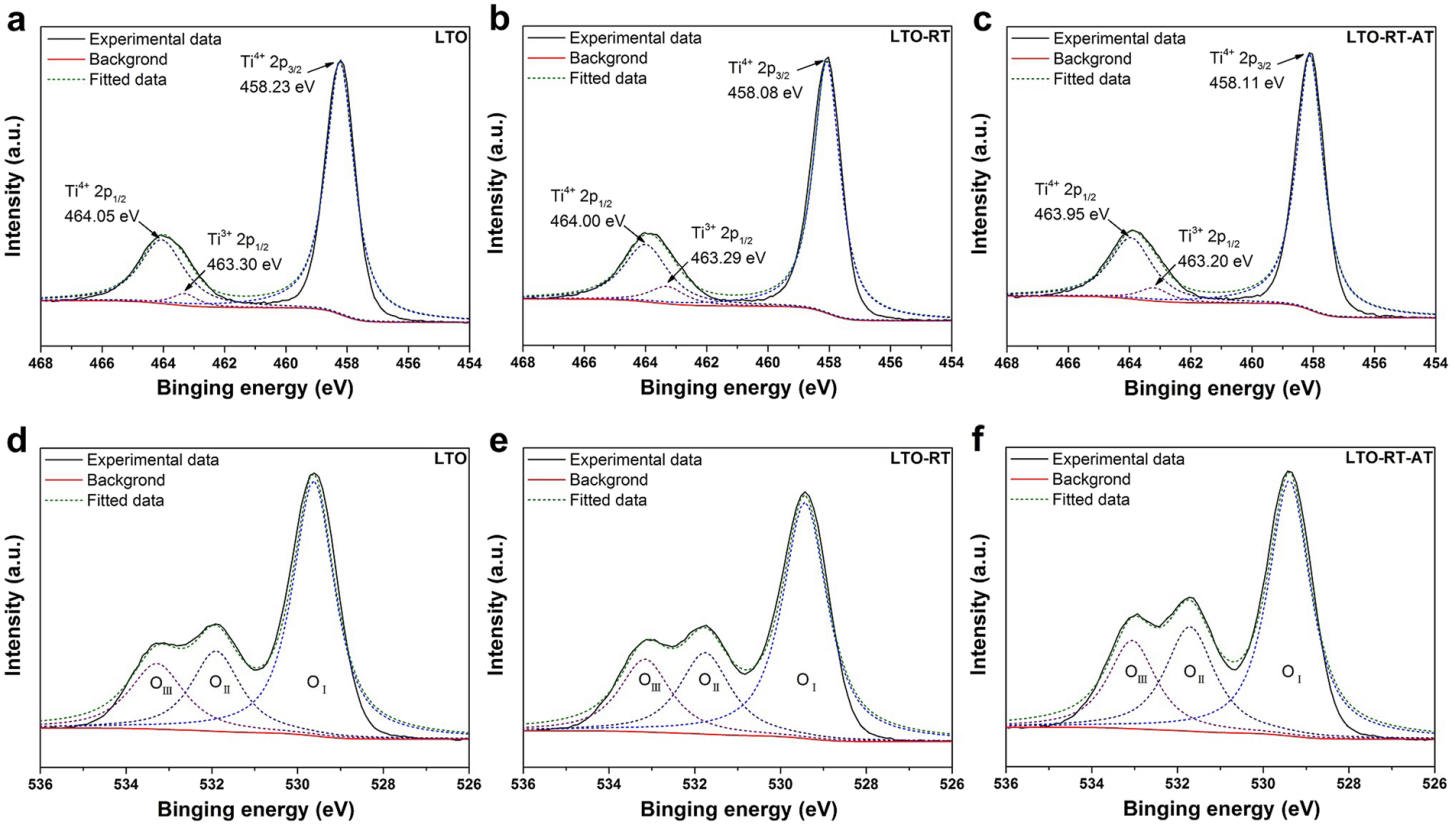
Ti 2p and O 1 s XPS spectra of (**a**,**d**) pure LTO, (**b**,**e**) LTO-RT and (**c**,**f**) LTO-RT-AT.

Additional information on the structure and oxidation states of pure LTO, LTO-RT and LTO-RT-AT was obtained from X-ray photoelectron spectroscopy (XPS) analysis. Figure 2a shows the high-resolution XPS spectra of Ti 2p for pure LTO. Two peaks at 464.05 and 458.23 eV correspond to the characteristic Ti 2p_1/2_ and Ti 2p_3/2_ peaks of Ti^4+^ ^26, 27^, respectively. In addition, a tiny peak at 463.30 eV can be attributed to Ti^3+^ ^26^. Note that almost all of the peak areas are assigned to Ti^4+^ and only a small peak area belongs to Ti^3+^ with a content of 3.02%. In comparison to pure LTO, the Ti 2p peaks of LTO-RT (Fig. 2b) and LTO-RT-AT (Fig. 2c) show a slight shift to lower binding energy, and the contents of Ti^3+^ increase to 6.31% for LTO-RT and 4.15% for LTO-RT-AT. These differences are probably ascribed to the increased oxygen vacancies in these LTO-TiO_2_ composites than those in pure LTO^28^. The incremental oxygen vacancies can decrease electron cloud density surrounding the titanium cores, therefore the electronic screening effect in the nanoparticles would be strengthened, causing the slightly decreased bonding energy values of the Ti 2p_1/2_ and Ti 2p_3/2_ signals^29^.

In order to further verify the above mentioned statement, the O 1 s spectra of the three samples are also shown in Fig. 2d–f. The O1s peaks can be fitted by three binding energy curves centered at ~529.5 (O_I_), 531.8 (O_II_), and 533.1 eV (O_III_), corresponding to the oxygen ions in the fully oxidized surrounding, the oxygen ions in oxygen deficient regions, and OH related species or the loosely bound oxygen on the surface from H_2_O^30^, respectively. The ratio of peak area (O_II_/O_I_ + O_II_), corresponding the relative quantity of the oxygen vacancies^31^, is increased from 26.83% for pure LTO to 27.81% for LTO-RT and 32.61% for LTO-RT-AT. The results of the O 1 s spectra agree well with the analysis of the Ti 2p spectra.

According to previous reports^32, 33^, the formation energy of oxygen vacancy significantly decreases in and around the grain boundary regions compared to the bulk and also the bulk-like structures. That is to say, grain boundary is beneficial for the formation of oxygen vacancy. Note that the grain size of LTO-RT-AT (26.5 nm), significantly smaller than that of pure LTO (32.6 nm) and LTO-RT (31.1 nm), resulting in a higher grain boundary density in the material. Therefore, we believe that the formation of more oxygen vacancies in LTO-RT-AT is closely related to its abundant grain boundaries. It has been reported that grain boundary can serve as reservoir for accumulating oxygen vacancies^34^, at the same time, oxygen vacancies can also stabilize the grain boundary structure^35^.

Such incremental oxygen vacancies are helpful for lithium ion insertion and electron transfer, which is beneficial to improving rate capability of the LTO-RT-AT. However, it is inexplicable that the LTO-RT-AT with the highest content of oxygen vacancies has a relatively lower content of Ti^3+^ in comparison with LTO-RT. Based on equilibrium of chemical valence, the content of oxygen vacancies is usually related to the content of Ti^3+^ in titanium-based oxides^24^. Therefore, the likely reason is the existence of lithium vacancies in the LTO-RT-AT. The oxygen vacancies and lithium vacancies charge compensate each other.

**Figure 3:**
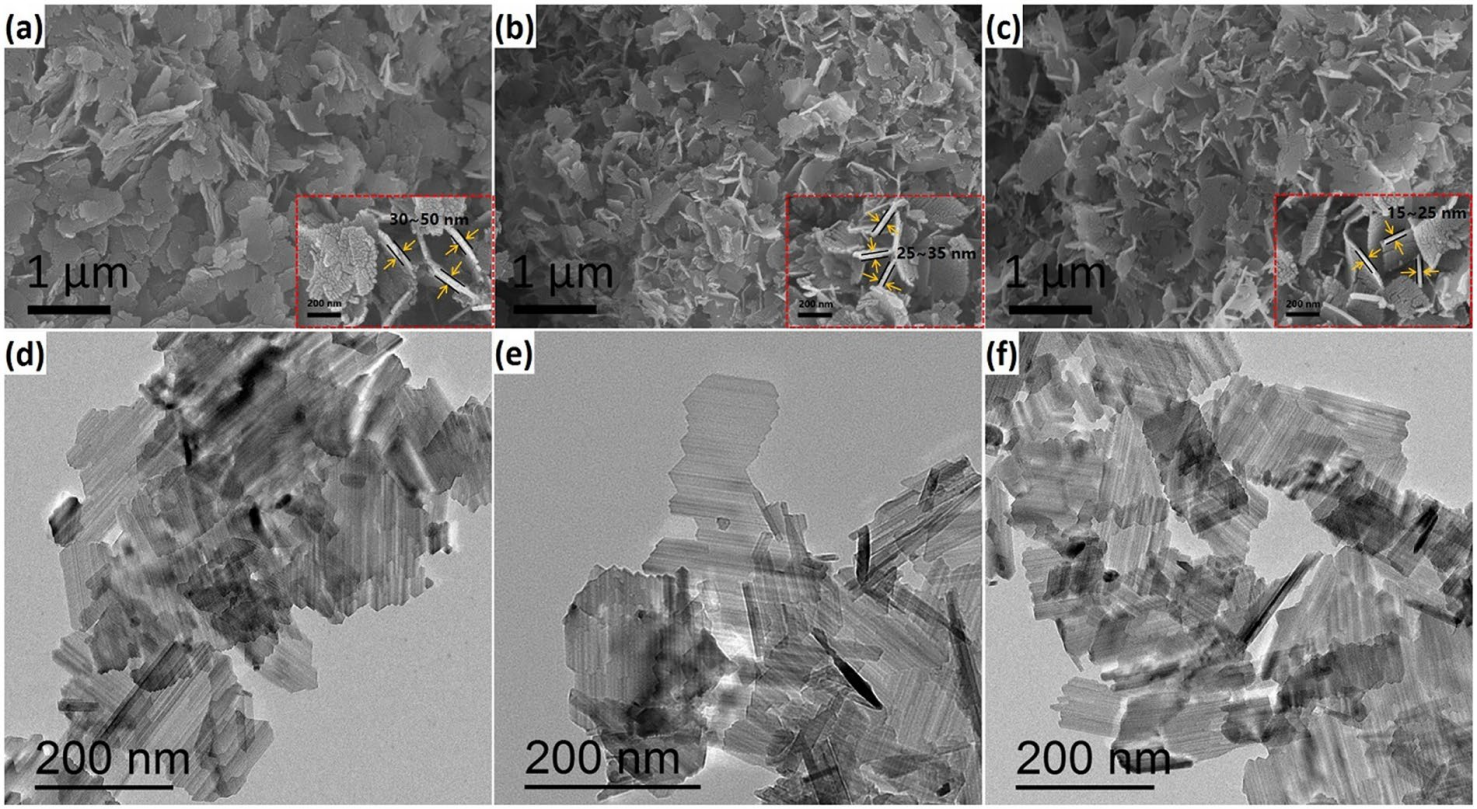
SEM and TEM images of (**a**,**d**) pure LTO, (**b**,**e**) LTO-RT and (**c**,**f**) LTO-RT-AT.

The morphology of pure LTO, LTO-RT and LTO-RT-AT were examined using a field emission scanning electron microscope (FESEM). As shown in Fig. 3, the three samples exhibit similar two-dimensional plate-like morphology, but the significantly different size, thickness and uniformity. The length and width of the pure LTO plates can reach over 1000 and 500 nm, respectively (Fig. 3a). By measuring the thickness at high magnification SEM (the insert of Fig. 3a), the thickness of the plates is 30~50 nm. In contrast, the LTO-RT (Fig. 3b) and LTO-RT-AT (Fig. 3c) plates exhibit better uniformity and smaller size. The thicknesses are 25~35 nm for LTO-RT (the insert of Fig. 3b) and 15~25 nm for LTO-RT-AT (the insert of Fig. 3c).

The transmission electron microscope (TEM) images show that these plates compose of smaller nanosheets, as shown in Fig. 3d–f. For pure LTO (Fig. 3d), the nanosheets also show the largest size (~500 nm in length and ~300 nm in width), while both LTO-RT and LTO-RT-AT nanosheets exhibit smaller size with length of ~200 nm and width of ~100 nm (Fig. 3e for LTO-RT; Fig. 3f for LTO-RT-AT). The above results clearly indicate that the TiO_2_ has important effects on the size of LTO nanosheets and contributes to suppressing their stacking, which may influence their electrochemical performance owing to the different path length for lithiumion transport^36, 37^.

**Figure 4:**
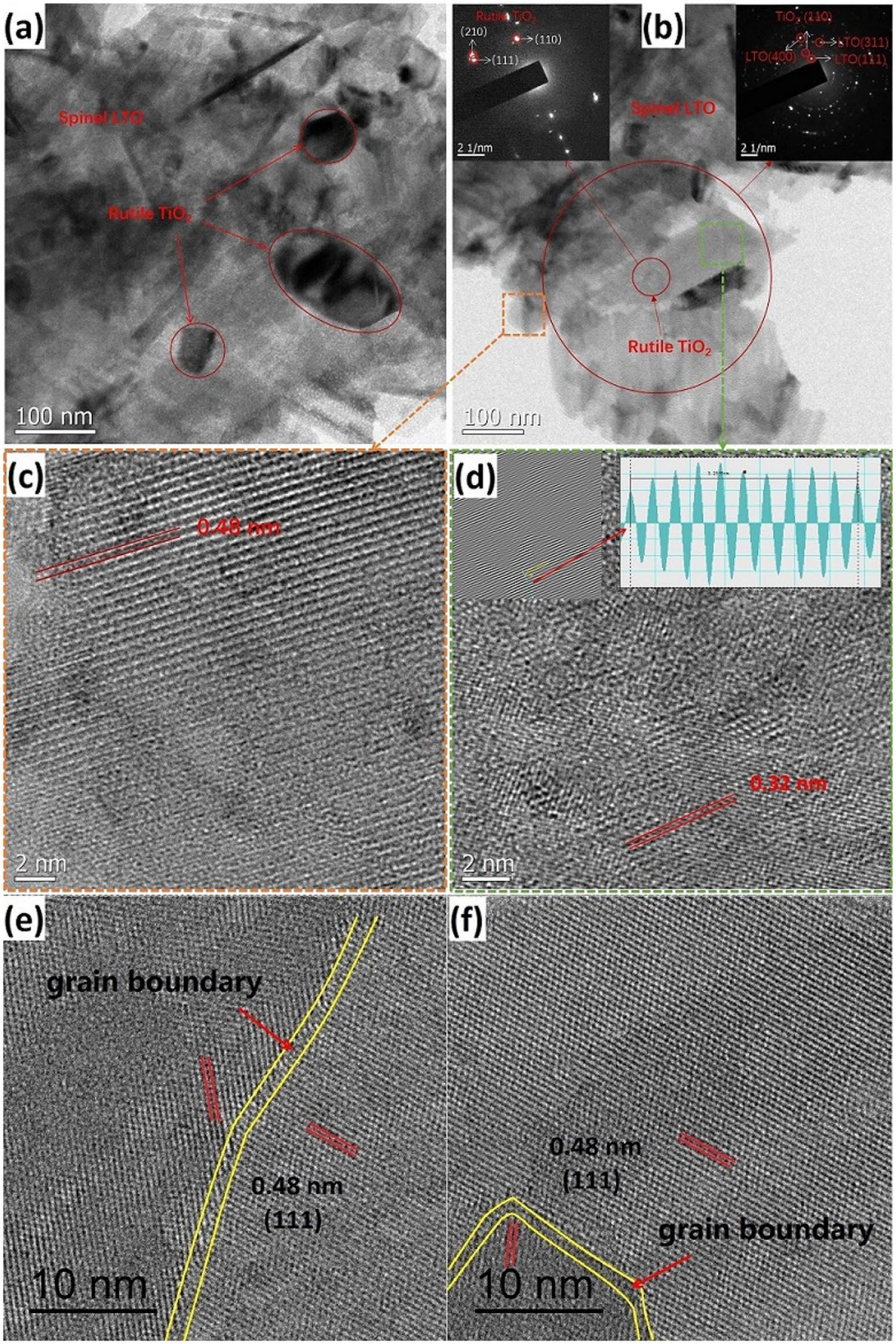
(**a**,**b**) TEM images and (**c**–**f**) HRTEM images of LTO-RT-AT. The insets in (**b**) show the SAED patterns. The yellow line areas in (**e**) and (**f**) show grain boundaries in the LTO-RT-AT.

More significantly, TEM analysis shows there are some smaller sizes polygons scattered on the surface of these LTO nanosheets in the LTO-RT-AT in Fig. 4a. The typical selected area electron diffraction (SAED) pattern of the well-defined polygons in the insert at the top left corner of Fig. 4b shows a set of sharp spots corresponding to rutile-TiO_2_ (110), (111) and (210) planes. Whereas the SAED pattern of the larger red circle area in Fig. 4b displays several marked ring patterns (the insert at the top right corner of Fig. 4b), corresponding to spinel LTO (111), (311), (400) and rutile-TiO_2_ (110) planes, which indicates the multiphase structure of the composite. The corresponding HRTEM images of LTO and rutile-TiO_2_ compositions are exhibited in Fig. 4c and d, respectively. The observed and calculated d-spacings from the HRTEM images are ~0.48 nm for LTO and 0.32 nm for rutile-TiO_2_, corresponding to the d-spacing of (111) facets of LTO and (110) facets of rutile-TiO_2_, respectively, suggesting the well-crystallized spinel phases and rutile phases in the LTO-RT-AT. However, the lattice fringes of anatase-TiO_2_ in the HRTEM images of LTO-RT-AT are not found, and this is due to its extremely low amount. In addition, many grain boundaries and lattice distortions (stacking fault) are clearly observed among the crystalline domains in LTO-RT-AT as indicated in Fig. 4e,f and S2 (Supporting Information), while few are seen in the pure LTO and LTO-RT.

**Figure 5:**
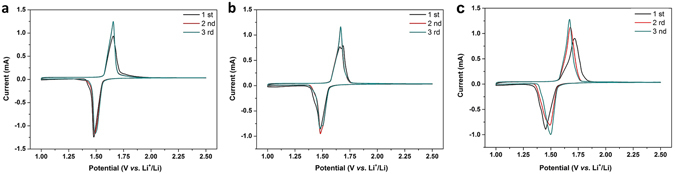
Cyclic voltammograms of (**a**) pure LTO, (**b**) LTO-RT and (**c**) LTO-RT-AT.

The electrochemical performance of pure LTO, LTO-RT and LTO-RT-AT was initially investigated by cyclic voltammetry (CV) at a scan rate of 0.1 mV s^−1^ in the potential range of 1.0~2.5 V (*vs*. Li/Li^+^), as shown in Fig. 5. It is clear that all the samples have a pair of redox peaks centered at ~1.5 V and ~1.65 V, corresponding to insertion and extraction of the lithium ions in the LTO lattice^38^. It is found that the peak potentials and shapes of LTO-RT-AT are slightly changed, and the potential differences between the anodic and cathodic potential peaks decrease with cycles, indicating that the polarization becomes weak gradually in LTO-RT-AT^39, 40^. In contrast, the peak potentials remain almost unchanged for pure LTO and LTO-RT. In addition, we do not find the characteristic redox peaks of TiO_2_ from CV results of the LTO-RT and LTO-RT-AT, suggesting that the electrochemical activity of TiO_2_ may be weak in this study, or it may be due to the low content of TiO_2_ in these LTO-TiO_2_ composites.

**Figure 6:**
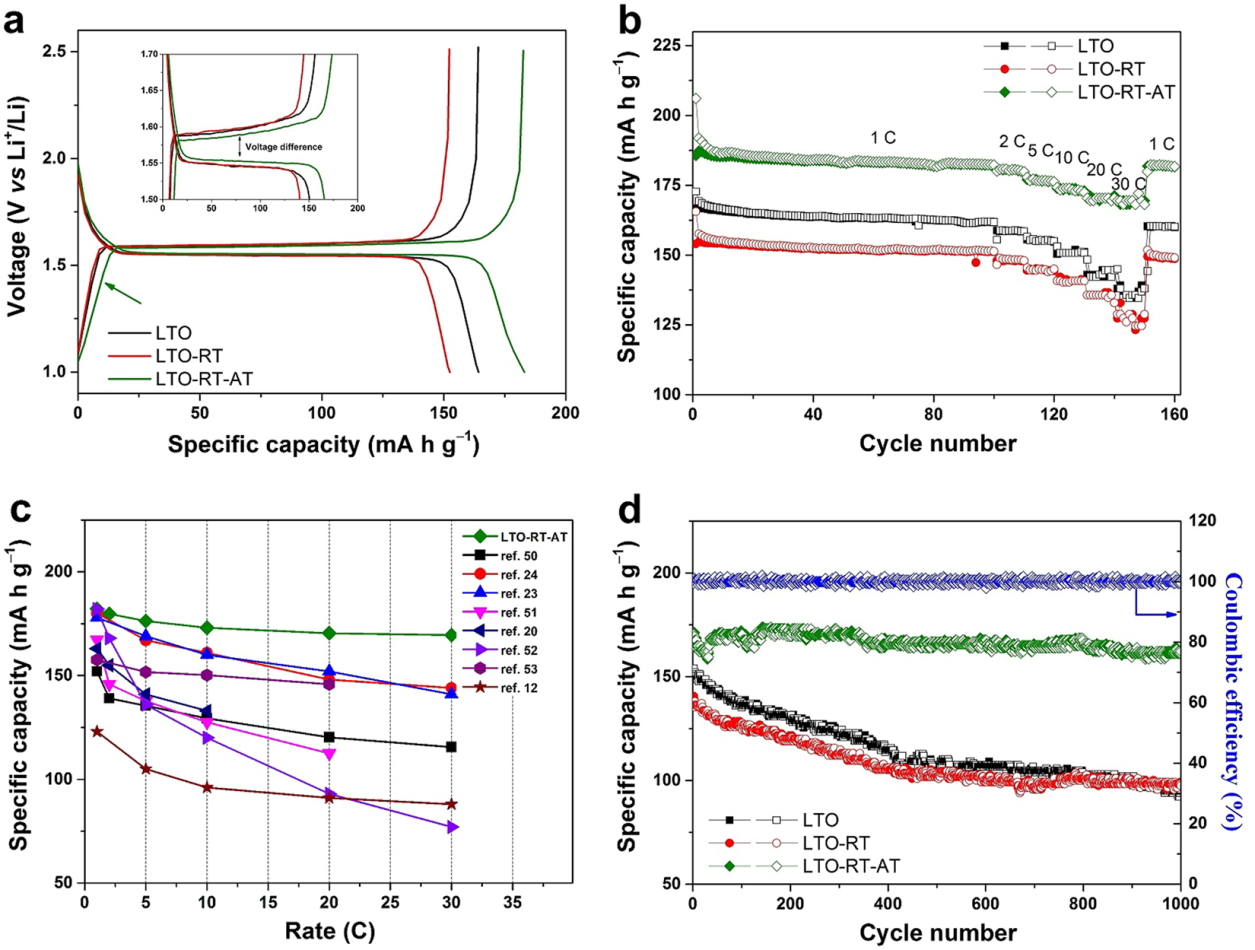
(**a**) Galvanostatic charge/discharge curves of pure LTO, LTO-RT and LTO-RT-AT, (**b**) cycle and rate performance at various rates of 1 to 30 C, (**c**) comparison of the rate capability of the LTO-RT-AT with other recently reported LTO-TiO_2_ composites, (**d**) long-term cycle performance and corresponding coulombic efficiency of LTO-RT-AT.

To demonstrate the potential application of LTO-RT-AT as LIBs anode materials, the galvanostatic measurements of test cells were performed at a current density of 1 C (1 C = 175 mA g^−1^) in the voltage range of 1.0 to 2.5 V (*vs*. Li/Li^+^). Figure 6a shows the steady-state charge/discharge curves of pure LTO, LTO-RT and LTO-RT-AT. The LTO-RT-AT exhibits the highest specific capacity and the smallest voltage difference between the charge and discharge (the insert of Fig. 6a), suggesting weaker polarization^41^. The finding agrees with the CV results well. The small polarization generally suggests a low internal resistance for electron and lithium ion transport in active materials in line with the excellent insertion/extraction behavior during the high rate cycle. The Nyquist plots and corresponding equivalent circuit based on electrochemical impedance spectroscopy (EIS) measurements are shown in Fig. S3 (Supporting Information). The weights and thicknesses of these tested electrodes are similar. EIS datas were collected after 3 cycles of each cell. It can be seen that the charge transfer resistance (*R*_*ct*_) of LTO-RT-AT is 23.69 Ω that is smaller than that of pure LTO (35.88 Ω) and LTO-RT (27.14 Ω).

In addition, a long sloped region (marked with arrow in Fig. 6a) between 1.55 and 1.0 V for the LTO-RT-AT is related to the pseudocapacitive lithium storage behavior^19^. To investigate the pseudocapacitive effect of the LTO-RT-AT electrode further, CV at different scan rates were performed and shown in Fig. S4 (Supporting Information). It is well known that the relation between peak current and scan rate reveals the different electrochemical reaction characteristics, including solid phase diffusion-controlled process or surface-confined charge-transfer process^19, 29^. For LTO-RT-AT (Fig. S4c), the lower potential peaks at 1.45, 1.40, 1.36, 1.29 and 1.19 V in the cathodic process appear with increasing the scan rate (marked with arrows) and proportionate with the square roots of the scan rates, suggesting an obviously pseudocapacitive effect in the LTO-RT-AT electrode. Whereas such peaks cannot be observed in pure LTO (Fig. S4a) and LTO-RT (Fig. S4b), indicating only the diffusion-controlled process in the two samples.

The above results indicate that the electrode reaction of LTO-RT-AT is controlled simultaneously by the diffusion-controlled and faradaic pseudocapacitive processes. It is well established that the pseudocapacitive effect can be induced due to the presence of abundant interfaces in the LTO-TiO_2_ composites, which is conducive to enhancing the specific capacity and rate capability of electrode materials. Similar results were also reported in these previous studies^29, 42–44^. By contrast, the pure LTO and LTO-RT have no obviously pseudocapacitive effect, suggesting their lower grain boundary density than the LTO-RT-AT.

Figure 6b exhibits the cycle profiles of the three samples at 1 C. The initial discharge and charge capacities of LTO-RT-AT are 206.1 and 185.6 mA h g^−1^, and this initial capacity loss is due to the irreversible lithium loss as a result of abundant structural defects and adsorbed trace amounts of water. The LTO-RT-AT also exhibits the good cycle performance and retains capacity up to 182.1 mA h g^−1^ after 100 cycles, which is much higher than that of pure LTO (161.7 mA h g^−1^) and LTO-RT (151.2 mA h g^−1^) tested in the same conditions.

To investigate the effect of rulite-TiO_2_ on the capacity of the LTO-TiO_2_ composites, pure rutile-TiO_2_ was synthesized without adding LiOH·H_2_O in the precursor solution. Figure S5a shows its XRD pattern (Supporting Information). The electrochemical result indicates that its specific capacity is very low (less than 20 mA h g^−1^) at a current density of 175 mA g^−1^, as shown in Fig. S5b. It is reasonable to believe that the rutile-TiO_2_ in LTO-RT and LTO-RT-AT can accommodate only small amounts of lithium ions and difficultly contribute to high capacity. This could be attributed to the highly anisotropic diffusion of lithium ions in rutile crystal structure hampering its performance^45^. Moreover, Liu *et al*.^46^ also found that the contribution of rulite-TiO_2_ to the overall capacity of the LTO-TiO_2_ composites is negligible at high current density.

Therefore, the ultrahigh specific capacity of LTO-RT-AT, even slightly higher than the theoretical value of LTO (175 mA h g^−1^), could be attributed to the following factors. Firstly, anatase-TiO_2_ has a higher specific capacity than LTO. Secondly, lithium ions can be stored in the structural defects of LTO-RT-AT. In consideration of the extremely low content of anatase-TiO_2_ (~0.456%) calculated according to the ratio of XRD peak area (anatase-TiO_2_/LTO-RT-AT), we believe the contribution from anatase-TiO_2_ to high capacity of LTO-RT-AT is very limited. The stepwise scanned XRD pattern and the areas of all the diffraction peaks of LTO-RT-AT are shown in Fig. S6 (Supporting Information). Therefore, the main reason for the ultrahigh specific capacity of LTO-RT-AT can be attributed to the lithium storage in the structural defects.

**Figure 7:**
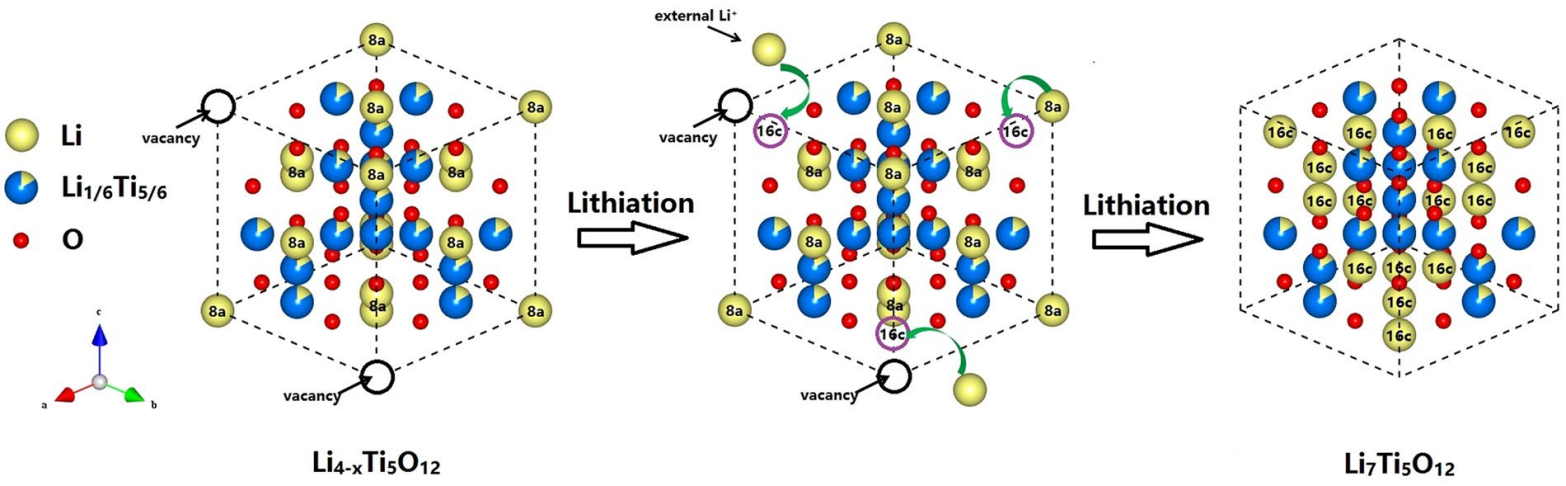
A schematic illustration of lithiation process from Li_4−x_Ti_5_O_12_ to Li_7_Ti_5_O_12_.

Benefiting from the high grain boundary density in LTO-RT-AT, a large number of lithium ions can be stored in these interfacial areas, leading to an obviously pseudocapacitive effect (Fig. S4c), increasing the specific capacity of the material. This is called as ‘interface lithium storage’ mechanism^47, 48^. Moreover, the lithium vacancies in the 8a sites of spinel LTO could allow more external Li ions insertion to 16c sites, as schematically illustrated in Fig. 7. Note that before lithiation, not all the tetrahedral 8a sites are occupied by Li, therefore the initial composition can be depicted as Li_4−x_Ti_5_O_12_. Upon lithiation, Li ions migrate from the tetrahedral 8a sites to the octahedral 16c sites, and external 3 + x Li ions insert to 16c sites to attain the final Li_7_Ti_5_O_12_ composition. It has been reported that holes can be trapped by lithium vacancies in lithium tetraborate (Li_2_B_4_O_7_) crystals^49^. A similar mechanism may also exist in the LTO-RT-AT. The lithium vacancies could also trap Li ions druing lithiation process. In light of this, the extra capacity beyond the theoretical value for LTO-RT-AT can be partly attributed to additional x Li ions.

Figure 6b also compares the rate capability of three samples measured at various current densities of 1 to 30 C. The capacities of the three samples decline slowly and show excellent rate capabilities from 1 to 10 C. When the current densities are above 20 C, nevertheless, the capacities of pure LTO and LTO-RT decrease significantly, whereas the capacity of LTO-RT-AT decreases much more slowly at the same rate. At the highest rate of 30 C, the LTO-RT-AT still delivers a capacity of 168.2 mA h g^−1^, which is much higher than that of pure LTO (134.6 mA h g^−1^) and LTO-RT (123.2 mA h g^−1^). A capacity of 181.6 mA h g^−1^ is retained for the LTO-RT-AT after 160 cycles when the current density recovers to 1 C. The remarkable rate capability of LTO-RT-AT can be attributed to its unique structural features. A large of structural defects significantly improved electrical conductivity and lithium ion diffusion kinetics properties of LTO-RT-AT electrode during the electrochemical reaction.

To be noted, the electrochemical performance of LTO-RT-AT is compared with other reported LTO-TiO_2_ composites in Fig. 6c, including LTO-TiO_2_ nanowire arrays^50^, copper-doped LTO-TiO_2_ nanosheets^24^, rutile-TiO_2_ coated LTO^23^, petal-like LTO-TiO_2_ nanosheets^51^, amorphous carbon coated LTO-TiO_2_^20^, hierarchical LTO-TiO_2_ microsphere^52^, LTO/rutile-TiO_2_ composite^53^ and LTO-TiO_2_-C nanocrystallines^12^. It is clear that the LTO-RT-AT possesses ultrahigh capacities and remarkable rate capability at various rates.

To explore the potential applications of LTO-RT-AT in electric vehicles (EV) and hybrid electric vehicles (HEV) which require both high-rate and long-life electrode materials, the fast charge and discharge performance at a high rate of 20 C for long-term cycles were measured and shown in Fig. 6d. It is observed that the LTO-RT-AT not only delivers an ultrahigh initial capacity of 171.3 mA h g^−1^, but also maintains long-term cycle stability with a capacity of 94.2% retention (161.3 mA h g^−1^) after 1000 cycles. The corresponding coulombic efficiency is held close to 100%, which is very important to the implementation and commercialization. In contrast, the capacities of pure LTO and LTO-RT decrease dramatically at the same condition. The lower capacities of 94.9 mA h g^−1^ for pure LTO and 98.3 mA h g^−1^ for LTO-RT are obtained after 1000 cycles, and the capacity retention rates of the two samples only remain at 62% and 69.9%, respectively.

## Discussion

In light of the above results, the ultrahigh reversible capacity, remarkable rate capability and long-term cycling stability of LTO-RT-AT can be mainly attributed to its abundant structural defects, including oxygen vacancies and grain boundaries. On the one hand, the oxygen vacancies are beneficial to improving the electrical conductivity of the electrode (increased carrier density) that facilitate the transport of charge carriers. On the other hand, the abundant grain boundaries not only provide more channels for lithium ion transport, enhancing lithium insertion/extraction kinetics, but also cause an obviously pseudocapacitive effect, significantly enhancing specific capacity and rate capability. Therefore, the unique structure of LTO-RT-AT is regarded as the crucial point, resulting in the superior electrochemical performance. This makes the reported LTO-RT-AT a promising new anode material for high-rate and long-life lithium-ion batteries and even for the potential applications in large scale energy storage.

## Methods

### Synthesis of LTO-AT-RT composite

In a typical synthesis, 20 mM of tetrabutyl titanate was added into 40 mL of anhydrous ethanol under mild stirring for 30 min. Meanwhile, 16.8 mM of LiOH·H_2_O was added into 40 mL of ultrapure water with stirring to allow for complete dissolution. The molar ratio of Li/Ti is 4.2:5. Then, the lithium hydroxide solution was dropwise added into the tetrabutyl titanate ethanol solution with vigorously stirring. The mixture solution was strongly stirred for 2 h to uniform the components. Subsequently, the solution was transferred to a 100 mL Teflon-lined autoclave, then sealed and heated at 180 °C for 24 h. The precipitates were centrifuged and washed thoroughly with ultrapure water and ethanol and then dried at 60 °C for 12 h under vacuum. The resulting product was calcined at 600 °C for 6 h in air to enhance crystallinity. Pure LTO and LTO-RT composite were also prepared using the same method, but the molar ratios of Li/Ti were 4.5:5 and 4:5, respectively.

### Materials Characterizations

The as-obtained samples were initially characterized by X-ray diffraction (XRD) using an X-ray diffraction analyzer with Cu-Kα radiation (D8-Discover, Bruker). Thermogravimetric (TG) and differential scanning calorimetry (DSC) were examined from the STA449 F3 thermal analyzer with a heating rate of 10 °C min^−1^ over the range 50~600 °C in an air atmosphere. X-ray photoelectron spectroscopy (XPS) measurements were performed on the product using a VG MultiLab 2000 system with a monochromatic Al Ka X-ray source (Thermo VG Scientific). The morphology of the samples were examined using a field emission scanning electron microscope (FESEM, Sirion, FEI). The detailed informations of the LTO-TiO_2_ composite were investigated using a high-resolution transmission electron microscope (HRTEM, Tecnai F20, FEI).

### Electrochemical Measurements

The electrochemical performance of the novel material was evaluated using CR2032-type coin cell. The working electrodes were fabricated by mixing the active materials with super P and polyvinylidene difluoride (PVDF) in a weight ratio of 75:15:10. Then, an appropriate amount of N-methylpyrrolidone (NMP) solvent was slowly added to the mixture to produce a slurry. The uniform slurry was coated onto a copper foil and dried at 120 °C for 24 h under vacuum. These electrodes were punched into the form of 13 mm diameter disks and assembled into half cells in an Ar-filled glove box. The mass loading of the active materials in each electrode was calculated to be ~2 mg cm^−2^ by weighing the mass discrepancy of the electrode and Cu foil. Lithium foils were used as the counter electrodes and the Celgard 2400 films were used as the separators. The commercial electrolyte was used which contained 1 M solution of LiPF_6_ in ethylene carbonate (EC) and dimethyl carbonate (DMC) with in a volume ratio of 1:1. The galvanostatic charge/discharge measurements were conducted using a LAND battery test system in the voltage range of 1.0 to 2.5 V (*vs*. Li/Li^+^) at different current densities. Cyclic voltammetry (CV) and electrochemical impedance spectroscopy (EIS) measurements were performed using a CHI660e electrochemical station.

## Electronic supplementary material

Supporting Information
