# Supplementary material for: Fabrication of Li4Ti5O12-TiO2 Nanosheets with Structural Defects as High-Rate and Long-Life Anodes for Lithium-Ion Batteries
Source: Sci Rep. 2017 Jun 7;7:2960. doi: 10.1038/s41598-017-03149-2 (PMC5462757; doi:10.1038/s41598-017-03149-2)
Supplement: Supplementary file 1 — Supporting Information [file 41598_2017_3149_MOESM1_ESM.doc]

Supporting Information

**Fabrication of Li_4_Ti_5_O_12_-TiO_2_ nanosheets with structural defects as high-rate and long-life anodes for lithium-ion batteries**

Hui Xu^1^, Jian Chen^1^*, Yanhuai Li^2^, Xinli Guo^1^, Yuanfang Shen^1^, Dan Wang^1^, Yao Zhang^1^, Zengmei Wang^1^

1 Jiangsu Key Laboratory of Advanced Metallic Materials, School of Materials Science and Engineering, Southeast University, Nanjing 211189, China

2 State Key Laboratory for Mechanical Behavior of Materials, Xi’an Jiaotong University, Xi’an 710049, Shaanxi, China

*E-mail: j.chen@seu.edu.cn


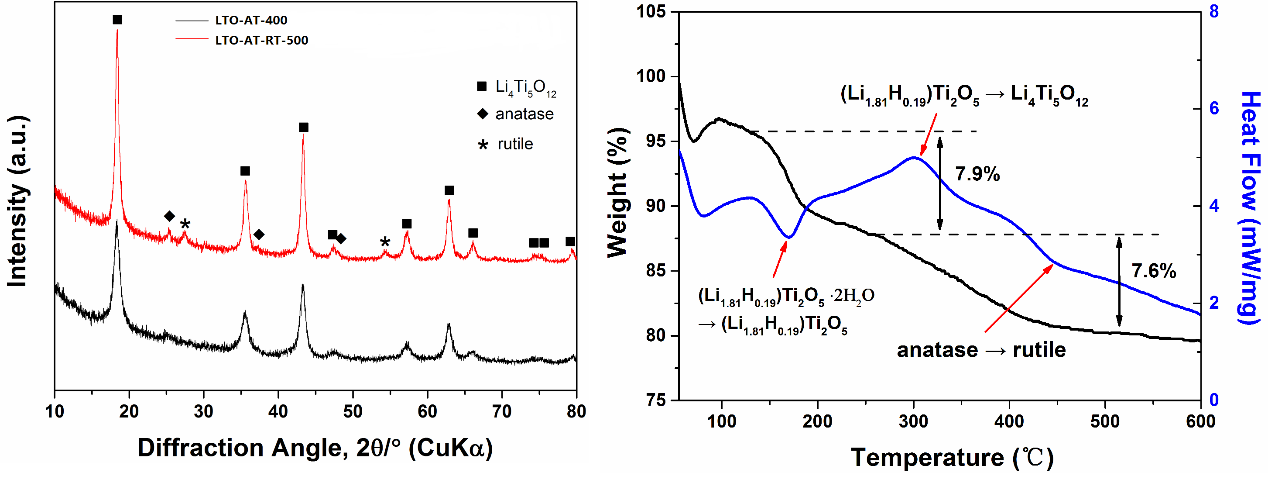


**Figure S1.** (a) XRD patterns of LTO-AT-400 and LTO-AT-RT-500, (b) TG-DSC traces of the precursors of LTO-AT-RT.


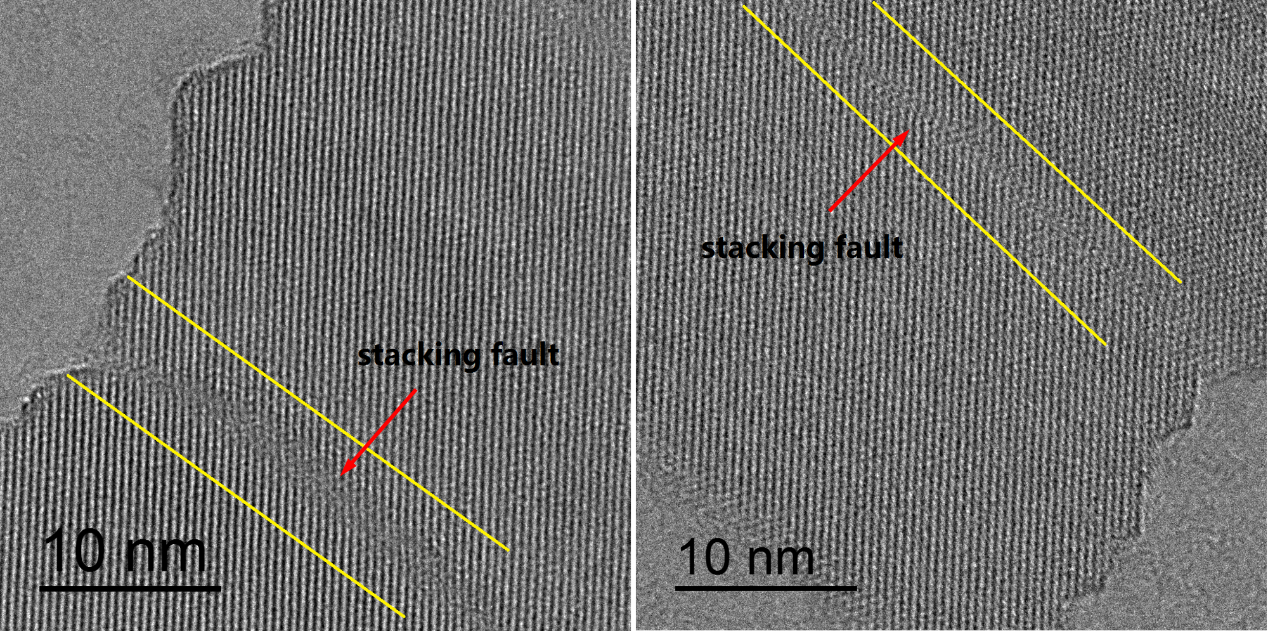


**Figure S2.** HRTEM images of LTO-RT-AT. The yellow line areas show lattice distortions (stacking fault) in the LTO-RT-AT.


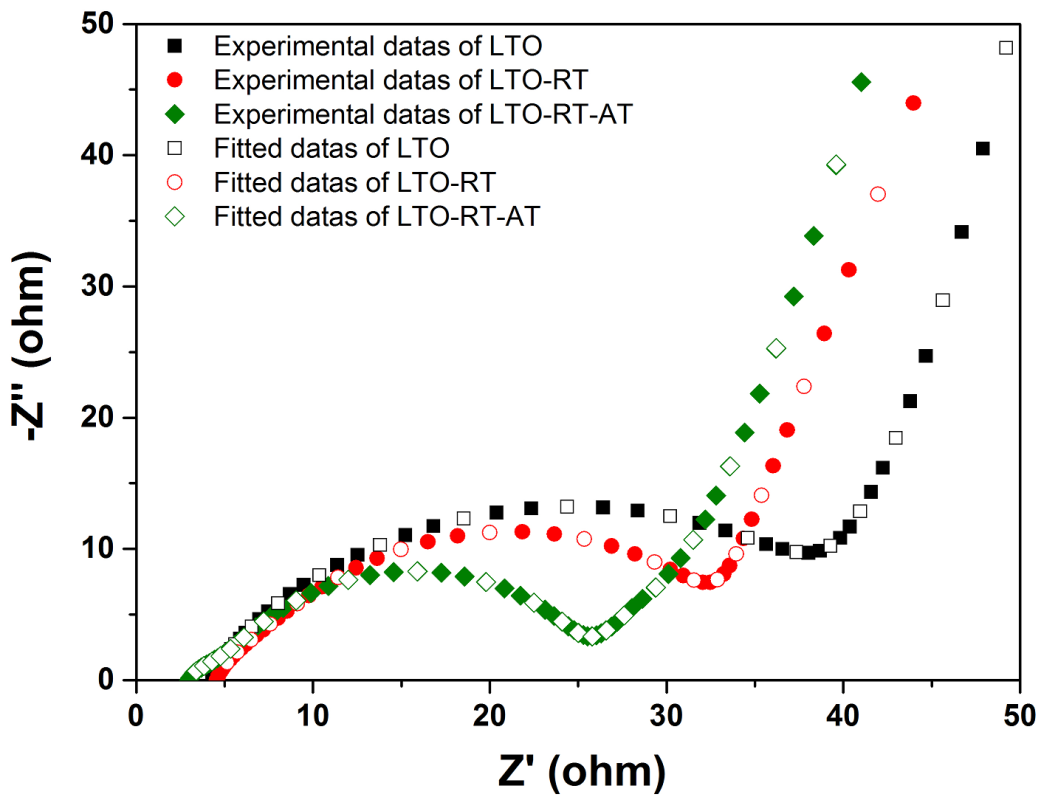


**Figure S3.** Electrochemical impedance spectra of pure LTO, LTO-RT and LTO-RT-AT.


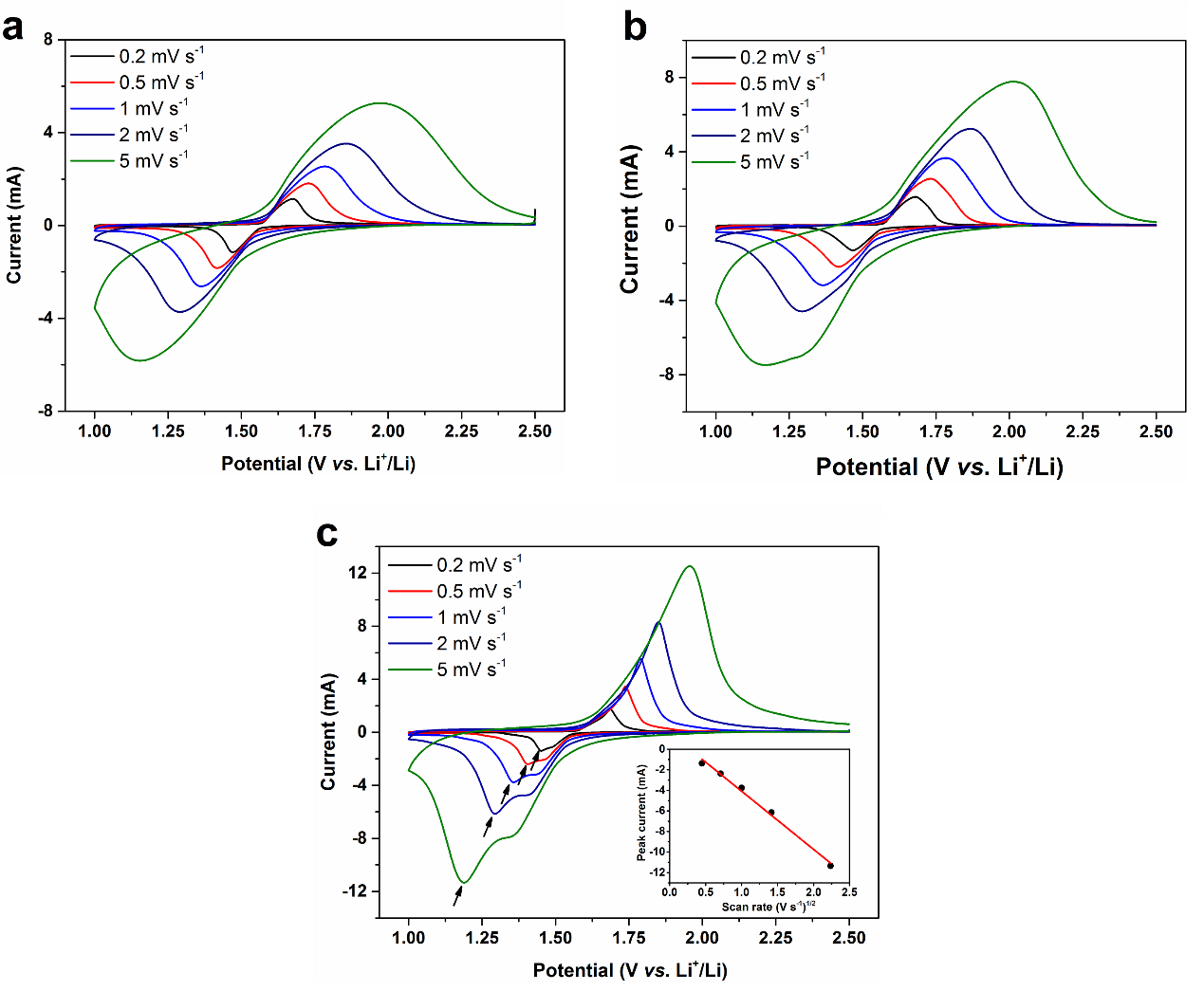


**Figure S4.** CV curves of (a) pure LTO, (b) LTO-RT and (c) LTO-RT-AT at different scan rates.


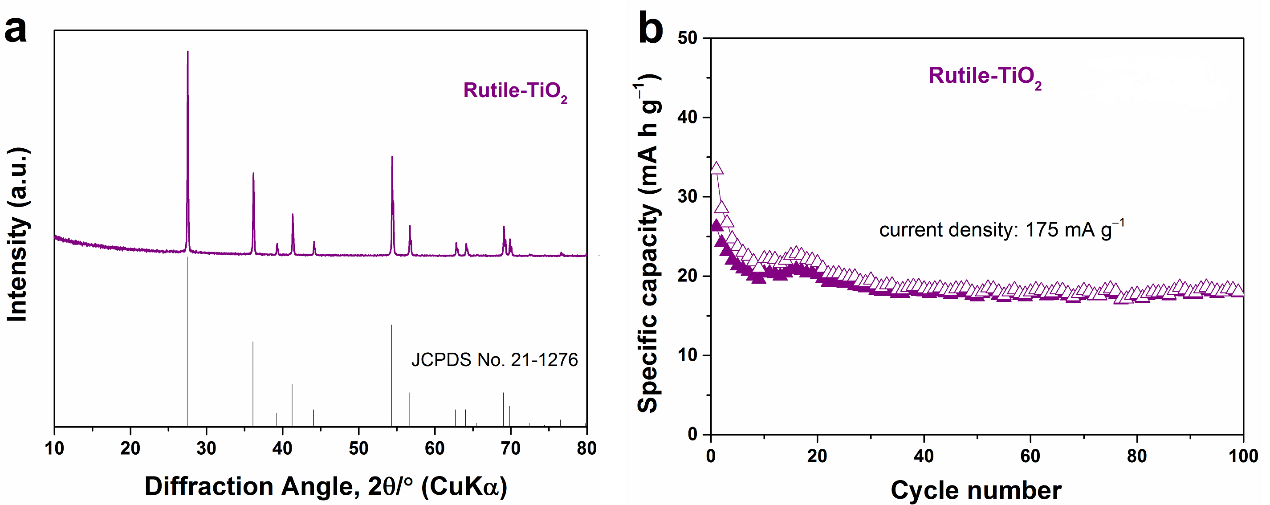


**Figure S5.** (a) XRD pattern of rutile-TiO_2_, (b) specific capacity of rutile-TiO_2_ tested at a current density of 175 mA g^−1^.


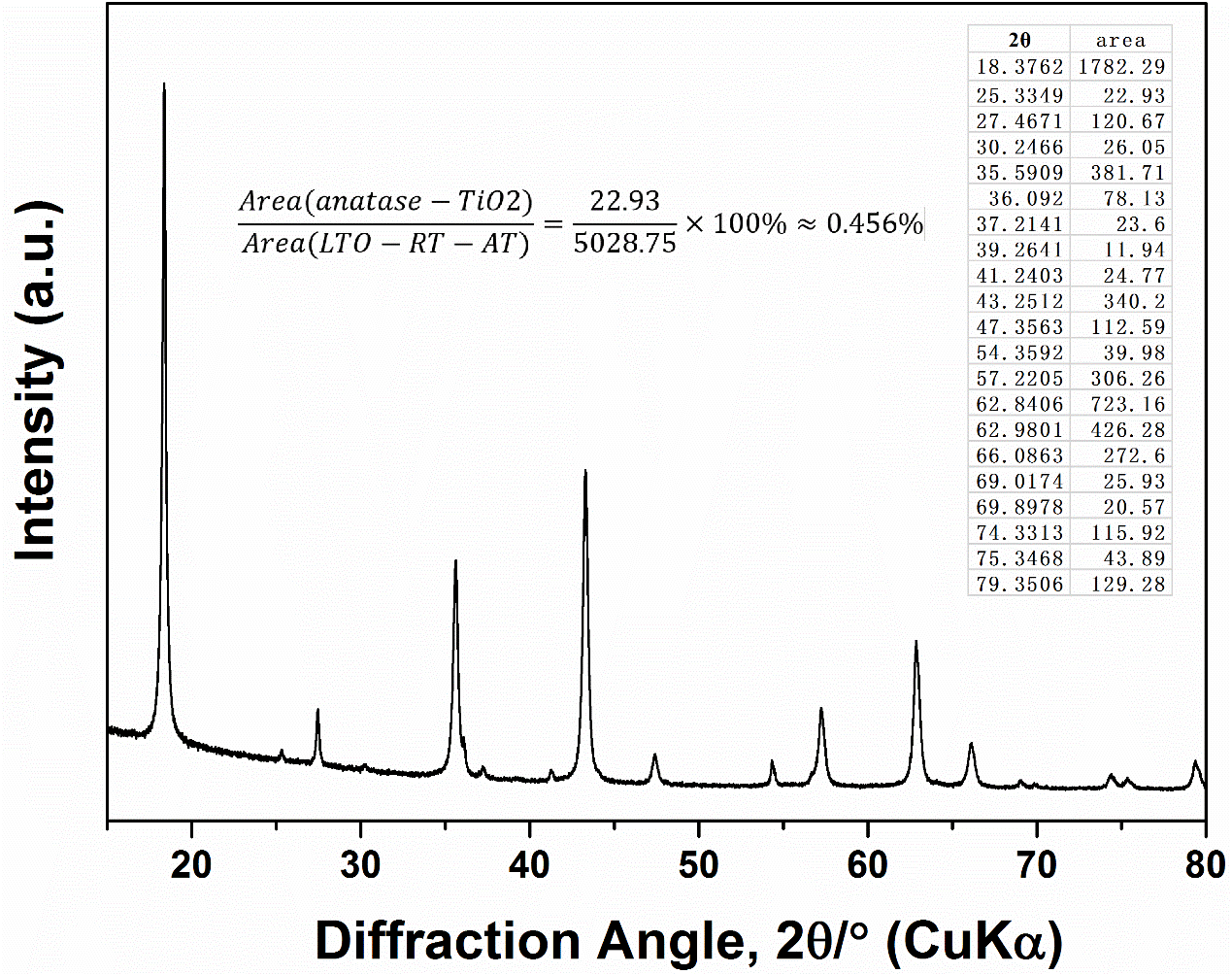


**Figure S6.** The stepwise scanned XRD pattern of LTO-RT-AT. The inset shows the areas of all the diffraction peaks.
